# Urban-Rural Differences in the Association Between Internet Use Trajectories and Depressive Symptoms in Chinese Adolescents: Longitudinal Observational Study

**DOI:** 10.2196/63799

**Published:** 2025-02-07

**Authors:** Yujie Liu, Xin Ge, Ying Wang, Xue Yang, Shangbin Liu, Chen Xu, Mi Xiang, Fan Hu, Yong Cai

**Affiliations:** 1 Public Health Research Center Tongren Hospital Shanghai Jiao Tong University School of Medicine Shanghai China; 2 School of Public Health Shanghai Jiao Tong University Shanghai China; 3 JC School of Public Health and Primary Care Faculty of Medicine Chinese University of Hong Kong Hong Kong China

**Keywords:** internet use, trajectory, depressive symptoms, adolescent, urban, rural

## Abstract

**Background:**

Internet use exhibits diverse trajectories during adolescence, which may contribute to depressive symptoms. Currently, it remains unclear whether the association between internet use trajectories and depressive symptoms varies between urban and rural areas.

**Objective:**

This study aimed to investigate the association between internet use trajectories and adolescent depressive symptoms and to explore variation in this association between urban and rural areas.

**Methods:**

This longitudinal study used 3-wave data from the 2014-2018 China Family Panel Study. Weekly hours of internet use and depressive symptoms were measured using self-reported questionnaires. Latent class growth modeling was performed to identify the trajectories of internet use. Multivariable logistic regressions were used to examine the association between internet use trajectories and depressive symptoms, stratified by rural and urban residence.

**Results:**

Participants were 2237 adolescents aged 10 to 15 years at baseline (mean age 12.46, SD 1.73 years). Two latent trajectory classes of internet use were identified: the low-growth group (n=2008, 89.8%) and the high-growth group (n=229, 10.2%). The high-growth group was associated with higher odds of depressive symptoms (OR 1.486, 95% CI 1.065-2.076) compared to the low-growth group. In the stratified analysis, the association between internet use trajectories and depressive symptoms was significant solely among rural adolescents (OR 1.856, 95% CI 1.164-2.959).

**Conclusions:**

This study elucidates urban-rural differences in the associations between trajectories of internet use and adolescent depressive symptoms. Our findings underscore the importance of prioritizing interventions for rural adolescents’ internet use behaviors to mitigate negative effects on their mental health.

## Introduction

Adolescence is a vulnerable period for developing depressive symptoms as a result of substantial biological and social transitions [1]. In China, the prevalence of depressive symptoms among adolescents has notably increased in recent decades [2,3]. A recent meta-analysis shows that approximately 19.9% of Chinese adolescents experience depressive symptoms [4]. The onset and persistence of depressive symptoms during this crucial developmental stage can result in a range of adverse outcomes in adulthood, such as functional impairment, suicidal self-harm, and mental health difficulties [5]. Therefore, early identification and intervention for depressive symptoms are imperative.

In recent years, the internet has become an essential tool for adolescents, widely used for information acquisition, social interaction, and entertainment [6]. According to the 2021 National Report on Internet Use by Minors, China has approximately 191 million internet users younger than 18 years, with a penetration rate of 96.8% [7]. Adolescents engage in various forms of internet use, including accessing online resources for educational purposes, such as completing school assignments [8]. Moreover, recreational and social activities, including video streaming, online gaming, and social networking, are also prevalent among this age group [9]. However, the widespread use of the internet has raised concerns about its effects on adolescent health [10-12]. Increasing evidence indicates a positive correlation between the duration of internet use and depressive symptoms in adolescents, with entertainment-related activities showing more pronounced negative effects [13-15]. In response, the Chinese government introduced regulations in 2019 to limit online gaming to 1.5 hours on weekdays and 3 hours on weekends to curb excessive internet use [16]. More recently, the Cyberspace Administration of China proposed further restrictions, recommending that adolescents aged 8 to 15 years be limited to 1 hour of daily mobile internet use, while those aged 16 to 18 years should not exceed 2 hours [17].

Lifestyle behaviors undergo dynamic changes during adolescence, characterized by a progressive rise in internet use as adolescents mature [18]. A longitudinal study of participants with a baseline age of 9.9 years reported significant increases in screen-based activities over 4 years: social media use rose from 0.5 to 2.5 hours per day, and video gaming from 0.9 to 2 hours per day, indicating an upward trajectory of internet use during early adolescence [19]. These evolving lifestyle patterns are well-established predictors of mental health outcomes [20]. Additionally, internet use may follow diverse subgroup trajectories within the overall developmental trend. For example, Yu and Park [21] used growth mixture modeling to classify South Korean adolescents into 4 distinct internet use trajectories: high stable, high quadratic, moderate stable, and low stable. Another study identified 2 subgroups: one with a sharp increase in internet use (drastically increasing group) and another with a more gradual rise (gradually increasing group), capturing varying rates of increase during adolescence [22]. These trajectories are associated with different risks for health outcomes [21,23]. However, whether internet use trajectories can predict depressive symptoms among adolescents remains unclear.

Furthermore, the impacts of internet use among adolescents may vary between rural and urban areas. Traditionally, rural adolescents have limited access to internet devices and engage in fewer online activities than their urban counterparts [24]. With the increasing penetration rates of the internet in recent years, the urban-rural gap has narrowed, with adolescents in rural areas experiencing a faster increase in screen time [25]. Consequently, the trajectories of internet use duration among rural and urban adolescents may differ over time. On the other hand, rural adolescents may face greater challenges due to a lack of parental supervision, as many of their parents migrate from rural to urban areas for work [7,26]. This absence of oversight could exacerbate the negative effects of excessive internet use. Therefore, it is essential to understand the varying associations between internet use trajectories in rural and urban adolescents and their mental health outcomes, which can offer empirical references for preventing adolescent depressive symptoms.

To date, the majority of research in China regarding the impact of adolescent internet use on mental health has focused on single regions, overlooking potential urban-rural differences across areas. This study seeks to address this knowledge gap by using nationally representative data for adolescents in 2014, 2016, and 2018. Our primary objective is to investigate the relationship between internet use trajectories throughout adolescence and depressive symptoms and to explore whether this relationship varies between urban and rural areas.

## Methods

### Study Sample and Procedure

This longitudinal study used data from the China Family Panel Study (CFPS), which covers populations from 25 provinces, municipalities, and autonomous regions in China. The CFPS uses a multistage sampling approach with implicit stratification based on urban-rural distinctions and regional socioeconomic status. Using a probability proportional to size sampling strategy, 32 streets and townships from Shanghai and 144 districts and counties from the other 24 provinces were selected as primary sampling units. Subsequently, 640 communities were randomly chosen from these units, and 25 households were systematically sampled from each community. The baseline survey in 2010 collected data from 33,600 adults and 8990 children across 14,960 families, who were invited to participate in follow-up surveys every 2 years. These surveys gathered comprehensive information on participants’ economic conditions, education, lifestyle, family relationships, and physical and mental health.

**Figure 1:**
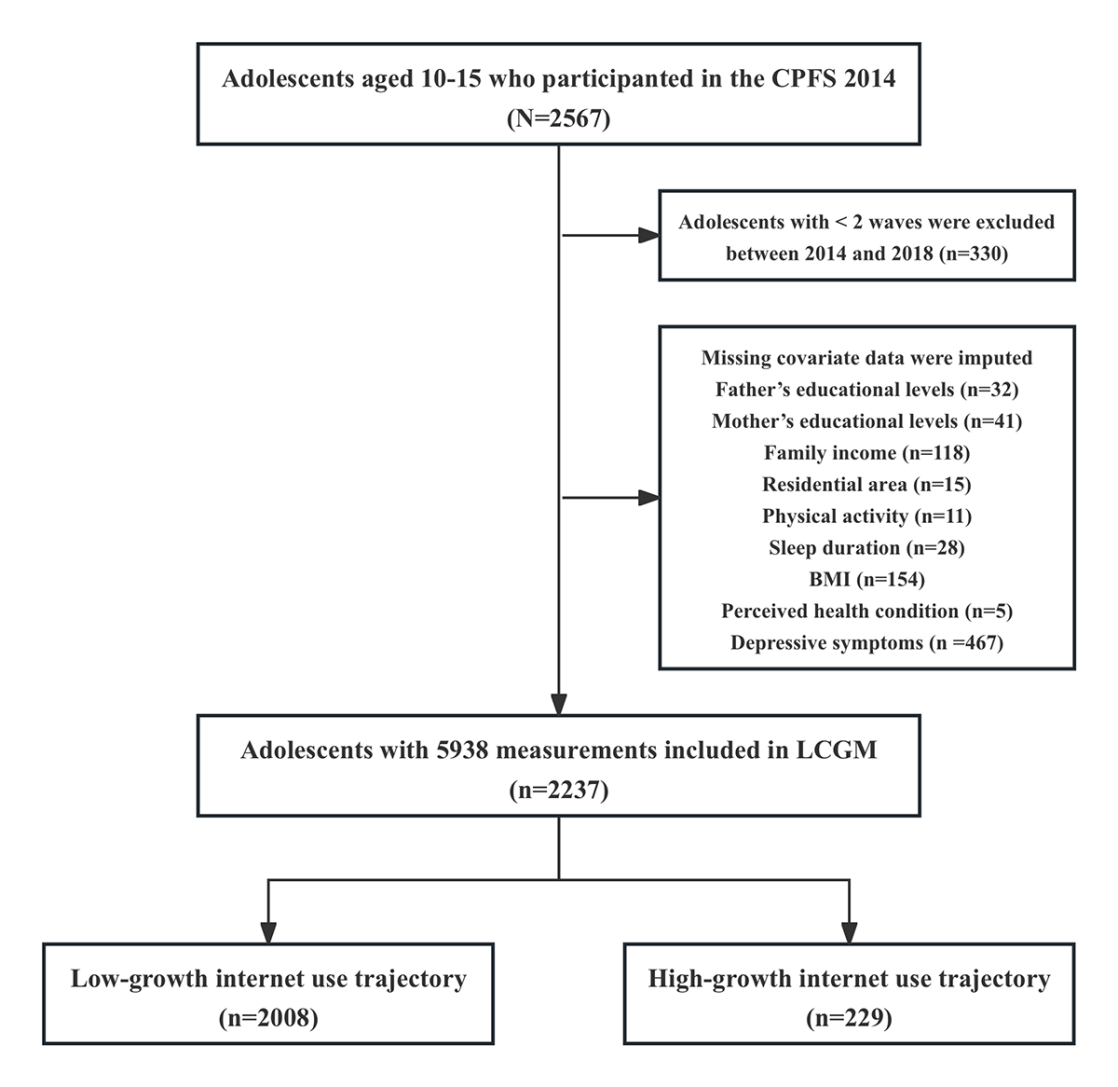
Flow chart of participation in this study. CFPS: China Family Panel Study; LCGM: latent class growth modeling.

For this study, we used 3 waves of data from the 2014-2018 cycle of the CFPS to ensure consistent measurement of internet use duration. In 2014, the sample included 2567 adolescents aged 10-15 years, of whom 1491 completed surveys in both the 2016 and 2018 cycles. By 2018, the participants’ age ranged from 14 to 19 years. To capture longitudinal developmental patterns, adolescents with self-reported data from at least 2 of the 3 waves were included, resulting in a final sample of 2237 participants. The flow of participants in this study is illustrated in Figure 1.

### Measurements

#### Internet Use

Internet use was measured using a single item: “In general, what is the total amount of time you spend online during your leisure time per week?” This variable is designed to measure leisure time spent online, with values ranging from 0 to 168 hours.

#### Depressive Symptoms

The Center for Epidemiologic Studies Depression (CESD) scale was used to assess depressive symptoms. The CESD evaluates a range of symptoms, including sadness, loss of interest, changes in appetite and sleep, difficulties in thinking or concentration, feelings of guilt, fatigue, movement changes, and suicidal ideation. Each item is rated on a 4-point scale (0=“not at all or less than 1 day in the past week” to 3=“nearly every day for 2 weeks”). To reduce respondent burden, in 2016, one-fifth of participants were randomly selected to complete the 20-item CESD (CESD-20), while the remaining four-fifths completed the 8-item version (CESD-8). All participants completed the CESD-8 in 2018 [27]. The CFPS considered the 2 groups in 2016 equivalent in depressive symptom distributions and used the equipercentile equating method to transform CESD-8 scores into CESD-20 scores, ensuring consistency and comparability across survey waves [27]. This approach aligns scores from the 2 versions by matching them to the same percentile rank [28]. The total CESD-20 score ranges from 0 to 60, with scores ≥16 indicating the presence of depressive symptoms based on established cutoffs [29]. CESD-8 and CESD-20 have demonstrated good psychometric properties in adolescents [30,31].

#### Covariates

Demographic variables included age (10-15 years in 2014), sex (male or female), parental educational levels (illiterate or semiliterate; primary school; junior high school; senior high school; college, university, or above), family income, residential area (rural or urban), and grandparental caregiving. Grandparental caregiving was determined based on responses to 2 questions: “Who takes care of the child during the day?” and “Who takes care of the child at night?” If either question was answered with “grandparents,” the child was classified as receiving grandparental care. Lifestyle and health-related variables included physical activity, sleep duration, BMI, and perceived health condition. Physical activity was assessed using a single item: “Including participation in physical education classes, what was the total amount of time you spent participating in physical activity in the past week?” Sleep duration was calculated as the time between falling asleep and waking up. BMI was determined by dividing objectively measured weight by height squared (kg/m^2^). Perceived health condition was assessed using a single item, “How do you perceive your health condition?” with responses ranging from 1 (“extremely healthy”) to 5 (“unhealthy”).

Baseline psychological distress was measured using the Kessler Psychological Distress Scale (K6). The K6 assesses the frequency of 6 symptoms: feeling nervous, hopeless, restless or fidgety, worthless, depressed, or that everything requires an effort [32]. Possible responses to the 6 items range from 0 (“none of the time”) to 4 (“all of the time”), yielding a total score of 0-24. The K6 has demonstrated good psychometric properties in adolescents [33,34].

### Statistical Analysis

The statistical analysis involved a 2-step procedure. In the first step, we conducted latent class growth modeling (LCGM) to determine trajectories of internet use across the 3 waves using Mplus (version 8.3; Muthén & Muthén). LCGM is a technique that identifies subgroups with distinct developmental patterns within a population over time [35]. With the 3-wave data, both linear and quadratic models were fitted to determine the optimal number of latent trajectory classes. The best-fitting model was determined based on the Akaike information criterion (AIC), Bayesian information criterion (BIC), sample size–adjusted BIC (aBIC), entropy, the Lo-Mendell-Rubin likelihood ratio test (LMR), and the bootstrapped likelihood ratio test (BLRT). Lower values of AIC, BIC, and aBIC indicate a better model fit. Higher entropy represents better classification accuracy. A significant *P* value (*P*<.05) for LMR and BLRT in a k-class model suggests that the model is better than the k–1 class model. Further, the sample size was taken into account, with at least 5% of the total population required in each class to define a meaningful latent entity [36].

In the second step, multivariable logistic regressions were conducted within the best-fitting model to examine the associations between internet use trajectories and distal outcomes. Each individual was assigned to their most likely class based on the highest posterior probability estimate, which was then used as the independent variable. The outcome variable was depressive symptoms in 2018. Three regression models were constructed. In model 1, adjustments were made for baseline K6 scores. Model 2 included additional demographic covariates, and model 3 further adjusted for lifestyle and health-related covariates. Stratified analysis was performed based on urban and rural classifications.

The Little missing completely at random (MCAR) test indicated that missing data for internet use followed an MCAR pattern (*χ*^2^_9_=6.616; *P*=.68). Therefore, missing data on internet use were handled using the robust full information maximum-likelihood estimation approach [37]. Missing data on covariates and the distal outcome were imputed using the *missForest* function in R (R Project for Statistical Computing), a method capable of handling both continuous and categorical variables [38]. We imputed missing values for the father’s educational level (n=32, 1.4%), mother’s educational level (n=41, 1.8%), family income (n=118, 5.4%), grandparental caregiving (n=13, 0.6%), residential area (n=15, 0.6%), physical activity (n=11, 0.5%), sleep duration (n=28, 1.2%), BMI (n=154, 7.1%), perceived health condition in 2014 (n=5, 0.2%), and depressive symptoms in 2018 (n=467, 21.5%).

### Ethics Approval

The Biomedical Ethics Review Committee of Peking University approved the study (IRB00001052-14,010). All eligible participants provided informed consent. The questionnaire was anonymous and did not collect any identifying information, such as name or address. Eligible participants were given CN ¥20-CN ¥60 (US $3.26-US $9.77) in cash as compensation.

## Results

### Descriptive Statistics

Table 1 presents the characteristics of the 2237 participants. The sample consisted of 1067 (47.7%) girls and 1170 (52.3%) boys, with a mean age of 12.46 (SD 1.73) years in 2014. Approximately half of the participants’ parents had a high school education or higher (father’s educational level: n=1187, 53.1%; mother’s educational level: n=938, 41.9%). The median annual household income was CN ¥39,000 (IQR CN ¥20,100-CN ¥61,200; US $3272-US $9962.60). A total of 60.4% (n=1351) of the participants were from rural areas, and 17.3% (n=386) were cared for by their grandparents. The median weekly hours of internet use were 0.0 (IQR 0.0-3.0) in 2014, 3.0 (IQR 0.0-8.0) in 2016, and 7.0 (IQR 2.0-20.0) in 2018. In 2018, the prevalence of depressive symptoms was 19.8% (n=444). CESD-20 scores in 2018 were positively correlated with K6 scores in 2014 (Spearman ρ=0.166; *P*<.001).

**Table 1:** Population characteristics.

Characteristics			Values (N=2237)
**Sex, n (%)**			
	Female	1067 (47.7)	
	Male	1170 (52.3)	
**Age (years)**			
	Mean (SD)	12.5 (1.7)	
	Range	10.0-15.0	
**Father’s education level, n (%)**			
	Illiterate/semiliterate	410 (18.3)	
	Primary school	640 (28.6)	
	Senior high school	775 (34.6)	
	Junior high school	261 (11.7)	
	College/university	151 (6.8)	
**Mother’s education level, n (%)**			
	Illiterate/semiliterate	686 (30.7)	
	Primary school	613 (27.4)	
	Senior high school	628 (28.1)	
	Junior high school	203 (9.1)	
	College/university	107 (4.8)	
**Annual family income (CN ¥; CN ¥1 = US $** **0.1628)**			
	Median (IQR)	39,000 (20,100-61,200)	
	Range	0-1,360,000	
**Residential area, n (%)**			
	Rural	1351 (60.4)	
	Urban	886 (39.6)	
**Grandparental caregiving, n (%)**			
	No	1851 (82.7)	
	Yes	386 (17.3)	
**Physical activity (** **hours/week)**			
	Median (IQR)	2.0 (0.0-4.0)	
	Range	0.0-56.0	
**Sleep duration (hours/day)**			
	Median (IQR)	9.0 (8.0-10.0)	
	Range	6.0-12.0	
**Internet use (2014) (hours/week)**			
	Median (IQR)	0.0 (0.0-3.0)	
	Range	0.0-84.0	
**Internet use (2016) (hours/week)**			
	Median (IQR)	3.0 (0.0-8.0)	
	Range	0.0-91.0	
**Internet use (2018) (hours/week)**			
	Median (IQR)	7.0 (2.0-20.0)	
	Range	0.0-144.0	
**BMI (kg/m^2^)**			
	Median (IQR)	17.8 (16.0-20.0)	
	Range	11.4-40.0	
**Perceived health condition, n (%)**			
	1	708 (31.6)	
	2	746 (33.3)	
	3	616 (27.5)	
	4	136 (6.1)	
	5	31 (1.4)	
**Kessler Psychological Distress Scale scores (2014)**			
	Median (IQR)	2.0 (0.0-4.0)	
	Range	0.0-21.0	
**Center for Epidemiologic Studies Depression scores (2018)**			
	Median (IQR)	30.0 (28.0-34.0)	
	Range	22.0-68.0	
**Depressive symptoms (2018), n (%)**			
	Nondepressed	1793 (80.2)	
	Depressed	444 (19.8)	

### LCGM Analysis

Table 2 presents the fit indices from the LCGM analysis for models with 1-4 trajectory classes. First, linear models were fitted. Reductions in AIC, BIC, and aBIC were observed across 1- to 4-class models. While both the LMR and BLRT statistics favored the 3-class model over the 2-class model, the latter exhibited higher entropy (0.909) with its smallest group >5%. Consequently, the 2-class model was selected as the best-fitting linear model. Subsequently, quadratic models were fitted, showing a similar pattern in fit indices to the linear models. The AIC, BIC, and aBIC values indicated an improvement in model fit upon adding the quadratic term. Therefore, the 2-class quadratic model was identified as the final optimal model of internet use trajectories.

**Figure 2:**
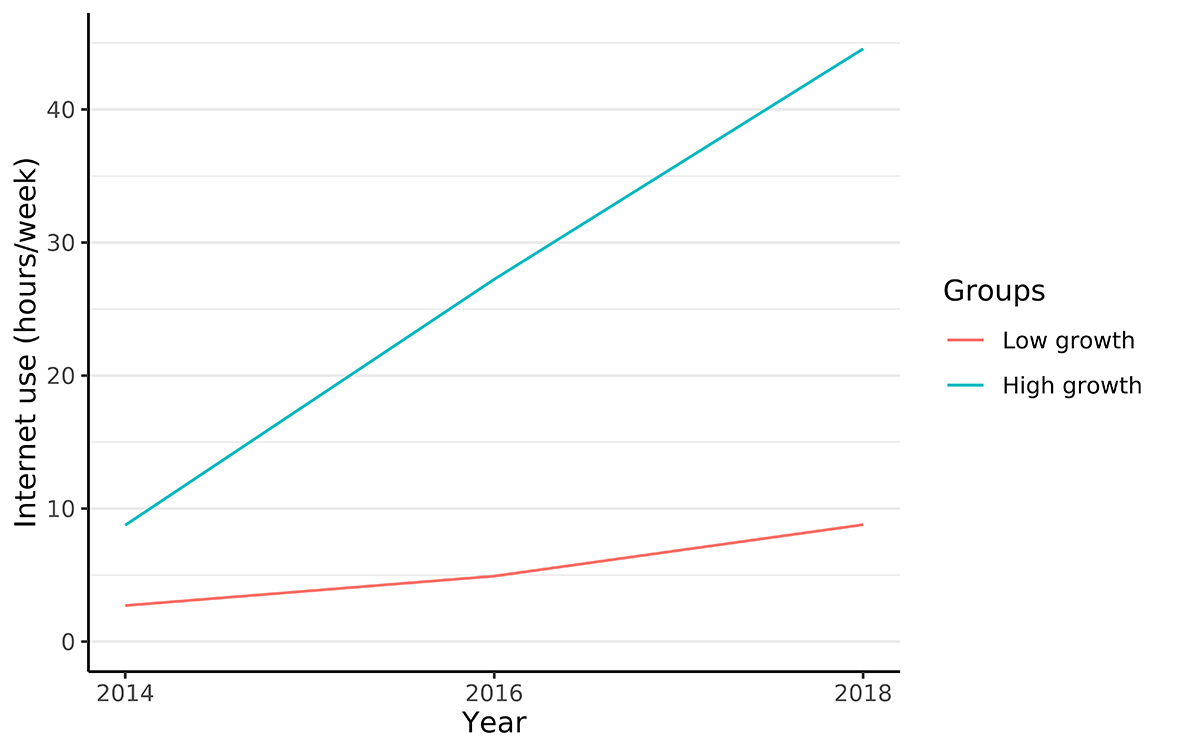
Latent trajectory classes of internet use from 2014 to 2018.

Figure 2 shows the 2 latent trajectory classes of internet use. Class 1 (89.8%) is labelled the “low-growth group,” representing the majority of adolescents who, on average, had relatively short internet use duration in 2014 (intercept=2.723; *P*<.001) and experienced gradual growth over 4 years (slope=1.080; *P*=.01; quadratic term=0.998; *P*<.001). Class 2 (10.2%) is labeled the “high-growth group,” characterized by initially longer average internet use duration (intercept=8.421; *P*<.001) and rapid growth until 2018 (slope=20.183; *P*=.008; quadratic term=–1.293; *P*=.74).

There were statistically significant differences across the trajectory classes in the distributions of age, parental educational level, annual family income, and residential area (Multimedia Appendix 1, Table S1). Specifically, urban participants were more likely to belong to the low-growth group compared to their rural counterparts.

**Table 2:** Model fit indices for latent class growth modeling.

Type of model and number of classes		AIC^a^	BIC^b^	aBIC^c^	Entropy	LMR^d^	BLRT^e^	Proportions^f^
**Linear model**								
	1	46,043.458	46,060.597	46,051.065	—^g^	—	—	1
	2	44,504.897	44,539.175	44,520.112	0.909	<.001	<0.001	0.892/0.108
	3	44,118.878	44,170.294	44,141.699	0.879	0.03	<0.001	0.840/0.129/0.031
	4	43,792.129	43,860.684	43,822.558	0.893	0.06	<0.001	0.806/0.141/0.034/0.019
**Quadratic model**								
	1	46,038.095	46,060.946	46,048.238	—	—	—	1
	2	44,495.849	44,541.552	44,516.135	0.914	0.01	<0.001	0.897/0.102
	3	43,469.206	43,537.761	43,499.635	0.893	0.007	<0.001	0.867/0.034/0.099
	4	42,837.724	42,929.13	42,878.295	0.883	0.20	<0.001	0.786/0.154/0.0345/0.025

^a^AIC: Akaike information criterion.

^b^BIC: Bayesian information criterion.

^c^aBIC: sample size–adjusted Bayesian information criterion.

^d^LMR: Lo-Mendell-Rubin likelihood ratio test.

^e^BLRT: bootstrapped likelihood ratio test.

^f^The values in this column represent the proportions of individuals in each class identified by latent class growth modeling.

^g^Not applicable.

### Association Between Trajectories and Depressive Symptoms

The percentage of adolescents with depressive symptoms in 2018 was 19.2% (385/2008) in the low-growth group and 25.8% (59/229) in the high-growth group. The difference in the percentages of depressive symptoms between the 2 groups was 6.6% (95% CI 1.1%-12%). Table 3 presents the results from multivariable logistic regressions examining the association between internet use trajectories and depressive symptoms. After adjusting for baseline psychological distress (model 1), the high-growth group was associated with higher odds of depressive symptoms compared to the low-growth group (OR 1.460, 95% CI 1.062-2.008; *P*=.02). The association remained significant after further adjustment for demographic, lifestyle, and health-related variables (model 3), with the high-growth group showing higher odds of depressive symptoms (OR 1.520, 95% CI 1.090-2.120; *P*=.01).

**Table 3:** Multivariable logistic regressions of internet use trajectory classes on depressive symptoms.

Rurality and internet use trajectory			Depressive symptoms, n (%)					Model 1^a^					Model 2^b^					Model 3^c^		
			Nondepressed		Depressed		OR (95% CI)			*P* value		OR (95% CI)			*P* value		OR (95% CI)			*P* value
**Overall (N=2237)**																				
	Low growth (n=2008)	1623 (80.8)		385 (19.2)		Ref			—^d^		—			—		—			—	
	High growth (n=229)	170 (74.2)		59 (25.8)		1.460 (1.062-2.008)			.02		1.501 (1.079-2.088)			.02		1.520 (1.090-2.120)			.01	
**Rural (n=1351)**																				
	Low growth (n=1241)	1009 (81.3)		232 (18.7)		Ref			—		—			—		—			—	
	High growth (n=110)	79 (71.8)		31 (28.2)		1.728 (1.111-2.686)			.02		1.874 (1.181-2.972)			.008		1.853 (1.162-2.955)			.01	
**Urban (n=886)**																				
	Low growth (n=767)	614 (80.1)		153 (19.9)		Ref			—		—			—		—			—	
	High growth (n=119)	91 (76.5)		28 (23.5)		1.192 (0.749-1.896)			.46		1.117 (0.686-1.819)			.66		1.124 (0.686-1.840)			.64	

^a^Model 1: Controlling for baseline psychological distress.

^b^Model 2: Controlling for baseline psychological distress, sex, age, parental educational level, annual family income, and grandparental caregiving.

^c^Model 3: Controlling for baseline psychological distress, sex, age, parental educational level, annual family income, grandparental caregiving, physical activity, sleep duration, BMI, and perceived health condition.

^d^Not applicable.

When stratified by residential area, the prevalence rates of depressive symptoms were 18.7% (232/1241) in the low-growth group and 28.2% (31/110) in the high-growth group among rural participants. The difference in the percentages of depressive symptoms between the 2 groups was 9.5% (95% CI 1.8%-17.2%). Results from the multivariable logistic regression indicated that the high-growth group was associated with higher odds of depressive symptoms among rural participants (OR 1.853, 95% CI 1.162-2.955; *P*=.01). Among urban participants, the prevalence rates were 19.9% (153/767) for the low-growth group and 23.5% (28/119) for the high-growth group. The difference in the percentages of depressive symptoms between the 2 groups was 3.6% (95% CI –4.2% to 11.4%). In the multivariable logistic regression, the association between internet use trajectories and depressive symptoms was not significant among urban participants (OR 1.124, 95% CI 0.686-1.840; *P*=.64).

### Sensitivity Analysis

We performed a sensitivity analysis to assess the robustness of our findings (Multimedia Appendix 1, Table S2). This analysis included additional adjustments for the CESD-20 scores in 2016. The results were consistent with the main analysis, with the association between internet use trajectories and depressive symptoms remaining significant among rural participants.

## Discussion

### Principal Findings

In this nationally representative study using data from the CFPS, 2 latent trajectory classes of internet use were identified among Chinese adolescents aged 10 to 19 years: a low-growth group (89.8%) and a high-growth group (10.2%). After delineating these distinct trajectories, we examined their associations with depressive symptoms and found that the high-growth group was associated with higher odds of depressive symptoms. Furthermore, our stratified analysis revealed variations in this association based on residential area. Specifically, while the association between trajectories of internet use and depressive symptoms remained significant among rural adolescents, it was not observed among their urban counterparts.

In this study, we observed a notable increase in the duration of internet use among adolescents over 4 years. This trend aligns with previous research indicating that adolescent internet use intensifies with age [18]. Zhu et al [39] found that adolescents tend to allocate more time to chatting or browsing the internet as they age. This increase in internet use among adolescents can be linked to the popularity of social media platforms, as well as their inclination toward social interaction and the development of virtual identities [39]. Furthermore, our analysis revealed heterogeneous developmental patterns within this trend, with 89.8% of adolescents exhibiting gradual growth in their internet use duration. In comparison, the remaining 10.2% increased their internet use to an average level approximately 5 times the baseline by the end of the follow-up. The diverse developmental patterns of these trajectories underscore the importance of early identification of excessive internet use. Understanding the contextual drivers of internet use behaviors is essential for altering the upward trajectory.

The high-growth group was associated with higher odds of depressive symptoms compared to the low-growth group. Our findings align with prior longitudinal research indicating that adolescent internet use can act as a risk factor for developing depressive symptoms [40]. However, previous studies have largely focused on static measures of internet use levels without examining how internet use evolves during adolescence. This study contributes to the existing evidence by demonstrating how trajectories of internet use can predict subsequent depressive symptoms. Various explanations may account for the observed association between internet use and depressive symptoms. For example, internet use may affect adolescents’ mental well-being by reducing time spent on physical activity and sleep [13,41]. However, even after adjusting for lifestyle factors, the association remained significant in this study, suggesting that time reallocation alone may not fully explain the relationship. Another possibility is that excessive internet use leads to reduced face-to-face interaction [42] and worsened academic performance [43], thereby increasing the risk of depressive symptoms among adolescents.

This study identified an urban-rural difference in how internet use trajectories were associated with depressive symptoms, specifically affecting adolescents residing in rural areas. Previous research has consistently highlighted the adverse effects of internet use on the mental well-being of rural adolescents in China [44,45]. For example, Zhou and Ding [46] examined the impacts of internet use across diverse adolescent groups and found stronger associations with mental health outcomes among those from less-developed regions. However, to our knowledge, this study is the first to reveal disparities between urban and rural adolescents in the impacts of long-term changes in internet use on depressive symptoms. Although rural adolescents showed a lower proportion of rapidly increasing internet use than their urban counterparts, this trajectory had a more pronounced effect on their mental health.

The primary factor driving urban-rural differences may be the insufficient parental supervision commonly experienced by rural adolescents. Only 38.3% of rural underage internet users report having their internet use regularly restricted by parents [7]. While grandparents in rural areas often provide caregiving in the absence of parents, their limited educational attainment and reliance on traditional caregiving practices may weaken their ability to effectively monitor adolescents’ internet use [47,48]. This lack of supervision can increase the likelihood of internet addiction, cyberbullying victimization, and engagement in high-risk online behaviors [49-51], potentially contributing to depressive symptoms. Additionally, individuals in rural areas with lower socioeconomic status often face limited social resources and reduced social support, which may amplify the association between internet use and depressive symptoms [52]. Rural schools, for example, tend to place less emphasis on internet literacy and education, resulting in lower awareness of healthy internet use practices [53,54]. The insufficient guidance may further heighten the risks to rural adolescents’ mental health.

### Implications

This study provides valuable insights into the distinct trajectories of internet use and their associations with adolescent depressive symptoms. The varying initial levels displayed by the trajectories in early adolescence highlight the critical need for early identification of excessive internet use. Given the demographic differences observed in this study, more attention is warranted to adolescents from lower socioeconomic backgrounds and rural areas.

The urban-rural divide in the association between internet use trajectories and depressive symptoms further emphasizes the importance of prioritizing interventions for rural adolescents. Previous research has shown that rural adolescents in China report higher levels of depressive symptoms compared to their urban peers [55]. Our findings suggest that rural adolescents are particularly vulnerable to the adverse effects of internet use, potentially widening existing mental health disparities between urban and rural areas. Effective interventions for rural adolescents should equip them with essential internet use skills, which can be fostered through enhanced parental supervision or integrated into school education programs. Additionally, parents should serve as positive role models by dedicating more time to engaging in outdoor activities with their children, providing alternatives to excessive screen time and encouraging a balanced lifestyle.

### Limitations

This study has several limitations. First, this study relied on self-reported measures of internet use and depressive symptoms. Although the CESD has demonstrated robust psychometric properties among adolescent populations [30,31], self-reported scales are subject to social desirability bias. Moreover, participants may inaccurately report their internet use due to recall bias, introducing potential measurement errors that could attenuate or exaggerate the observed associations between internet use and depressive symptoms. Future research could benefit from incorporating objective measures of internet use and clinically validated tools for assessing depressive symptoms to enhance the reliability of findings. Second, the CFPS did not assess depressive symptoms in 2014. However, baseline psychological distress was controlled using the K6 scale, which was positively correlated with CESD-20 scores in 2018. Furthermore, we found that additional adjustments for CESD-20 scores in 2016 did not alter the association between internet use trajectories and depressive symptoms, indicating the robustness of our findings. Third, the assessment of internet use duration was based on a single question, limiting insights into the specific online activities adolescents engaged in. Future research could consider incorporating comprehensive measures of diverse online activities. Fourth, our sample was limited to adolescents in China. Disparities in health issues between rural and urban areas may exhibit different patterns among adolescents from other countries. Therefore, our findings may not be generalizable to a global population. Finally, in this study, the sample size was relatively small, particularly when considering the urban-rural stratification. Future research should aim to use larger, nationally representative datasets to further explore variations in the effects of adolescent internet use between urban and rural regions.

### Conclusion

This study elucidates urban-rural differences in the associations between internet use trajectories and adolescent depressive symptoms. Over 4 years, we observed diverse trajectories of internet use among adolescents, with one subgroup showing a rapid increase in internet use duration that was associated with higher odds of depressive symptoms. Further stratified analysis revealed that this association was significant only among rural adolescents. These findings offer valuable insights into regional disparities in the impacts of adolescent internet use. Tailored intervention strategies are needed to address the challenges faced by rural adolescents, thereby mitigating the negative effects of excessive internet use on their mental health.

## Appendix

Multimedia Appendix 1 Tables S1 and S2.

## Abbreviations

aBIC: sample size–adjusted Bayesian information criterion
AIC: Akaike information criterion
BIC: Bayesian information criterion
BLRT: bootstrapped likelihood ratio test
CESD: Center for Epidemiologic Studies Depression Scale
CFPS: China Family Panel Study
K6: Kessler Psychological Distress Scale
LCGM: latent class growth modeling
LMR: Lo-Mendell-Rubin likelihood ratio test
MCAR: missing completely at random

## Data Availability

The data that support the findings of this study are available from the Peking University Open Research Data Platform [56].
