# Supplementary material for: Urban-Rural Differences in the Association Between Internet Use Trajectories and Depressive Symptoms in Chinese Adolescents: Longitudinal Observational Study
Source: J Med Internet Res. 2025 Feb 7;27:e63799. doi: 10.2196/63799 (PMC11845883; doi:10.2196/63799)
Supplement: Multimedia Appendix 1 [file jmir_v27i1e63799_app1.docx]

**Table S1. Characteristics of participants by internet use trajectory classes**

|  | **Internet use trajectory class^a^** | | Statistic | *P* value |
| --- | --- | --- | --- | --- |
|  | Low growth (N = 2008) | High growth (N = 229) |  |  |
| **Gender** |  |  |  |  |
| Female | 967 (48.2%) | 100 (43.7%) | 1.661 (1)^b^ | 0.20 |
| Male | 1041 (51.8%) | 129 (56.3%) |  |  |
| **Age** | 12.4 ± 1.73 | 13.3 ± 1.55 | -8.021 (2235)^c^ | <.001 |
| **Father's education level** |  |  |  |  |
| Illiterate/semi-literate | 382 (19.0%) | 28 (12.2%) | 10.538 (4)^b^ | 0.03 |
| Primary school | 576 (28.7%) | 64 (27.9%) |  |  |
| Senior high school | 685 (34.1%) | 90 (39.3%) |  |  |
| Junior high school | 226 (11.3%) | 35 (15.3%) |  |  |
| College/university | 139 (6.9%) | 12 (5.2%) |  |  |
| **Mother's education level** |  |  |  |  |
| Illiterate/semi-literate | 644 (32.1%) | 42 (18.3%) | 24.006 (4)^b^ | <.001 |
| Primary school | 543 (27.0%) | 70 (30.6%) |  |  |
| Senior high school | 551 (27.4%) | 77 (33.6%) |  |  |
| Junior high school | 171 (8.5%) | 32 (14.0%) |  |  |
| College/university | 99 (4.9%) | 8 (3.5%) |  |  |
| **Family annual income** | 38.8 (20.0–60.6) | 42.6 (22.7–67.2) | -1.622^d^ | 0.11 |
| **Residential area** |  |  |  |  |
| Rural | 1241 (61.8%) | 110 (48.0%) | 16.290 (1)^b^ | <.001 |
| Urban | 767 (38.2%) | 119 (52.0%) |  |  |

^a^ Data are presented as mean ± standard deviation or Median (Q1–Q3 quartiles) for continuous variables and n (%) for categorical variables.

^b^ Chi-square statistic and degrees of freedom

^c^ T-test statistic and degrees of freedom

^d^ Mann-Whitney U test statistic

**Table S2. Sensitivity analysis on associations between internet use trajectory classes and depressive symptoms**

| Internet use trajectory | Model 1 | | Model 2 | | Model 3 | |
| --- | --- | --- | --- | --- | --- | --- |
|  | OR (95%CI) | *P* value | OR (95%CI) | *P* value | OR (95%CI) | *P* value |
| **Overall (N=2237)** | | | | | | |
| Low growth | Ref | | | | | |
| High growth | 1.402 (1.012–1.943) | 0.04 | 1.433 (1.022–2.008) | 0.04 | 1.454 (1.035–2.042) | 0.03 |
| **Rural (N=1351)** | | | | | | |
| Low growth | Ref | | | | | |
| High growth | 1.665 (1.059–2.620) | 0.03 | 1.787 (1.112–2.870) | 0.02 | 1.776 (1.100–2.865) | 0.02 |
| **Urban (N=886)** | | | | | | |
| Low growth | Ref | | | | | |
| High growth | 1.130 (0.703–1.818) | 0.61 | 1.069 (0.651–1.757) | 0.79 | 1.078 (0.652–1.781) | 0.77 |

Model 1: Controlling for 2014 psychological distress and 2016 depressive symptoms.

Model 2: Controlling for 2014 psychological distress, 2016 depressive symptoms, sex, age, parental educational level, family annual income, and grandparental caregiving.

Model 3: Controlling for 2014 psychological distress, 2016 depressive symptoms, sex, age, parental educational level, family annual income, grandparental caregiving, physical activity, sleep duration, BMI, and perceived health condition
